# Supplementary material for: A retrospective study: does upper airway morphology differ between non-positional and positional obstructive sleep apnea?
Source: PeerJ. 2017 Oct 13;5:e3918. doi: 10.7717/peerj.3918 (PMC5642243; doi:10.7717/peerj.3918)
Supplement: Supplemental Information 1 — SPL, soft palate length; CD, coronal diameter; AHI, apnea hyponea index. [file peerj-05-3918-s001.docx]

Supplementary

**Table 1**. Analysis of the forward logistic regression models for predicting NPOSA.

| indicators | OR | 95%CI | p |
| --- | --- | --- | --- |
| SPL(mm) | 1.205 | 1.055-1.377 | 0.006 |
| CD of the narrowest level of glossopharygeal | 0.881 | 0.793-0.980 | 0.020 |
| Age | 1.205 | 1.005-1.130 | 0.033 |

SPL= soft palate length; CD= coronal diameter; AHI = apnea hyponea index.
